# The association between dietary pattern and visceral adiposity index, triglyceride-glucose index, inflammation, and body composition among Iranian overweight and obese women

**DOI:** 10.1038/s41598-023-39653-x

**Published:** 2023-08-13

**Authors:** Fatemeh Gholami, Zahra Karimi, Mahsa Samadi, Neda Sovied, Mir Saeid Yekaninejad, Seyed Ali Keshavarz, Gholamali Javdan, Niki Bahrampour, Alexei Wong, Cain C. T. Clark, Khadijeh Mirzaei

**Affiliations:** 1 Department of Community Nutrition, School of Nutritional Sciences and Dietetics, Tehran University of Medical Sciences (TUMS), P.O. Box: 14155-6117, Tehran, Iran; 2 Department of Epidemiology and Biostatistics, School of Public Health, Tehran University of Medical Science, Tehran, Iran; 3 Department of Clinical Nutrition, School of Nutritional Sciences and Dietetics, Tehran University of Medical Sciences, Tehran, Iran; 4 Food Health Research Center, Hormozgan University of Medical Sciences, Bandar Abbas, Iran; 5 Department of Nutrition, Science and Research Branch, Islamic Azad University (SRBIAU), Tehran, Iran; 6 Department of Health and Human Performance, Marymount University, Arlington, VA USA; 7 Centre for Intelligent Healthcare, Coventry University, Coventry, CV1 5FB UK

**Keywords:** Diseases, Health care

## Abstract

The aim of the present study was to investigate the association between dietary patterns, derived through latent class analysis (LCA), with visceral adiposity index (VAI), Triglyceride-Glucose Index (TyG), inflammation biomarkers, and body composition in overweight and obese Iranian women. For this cross-sectional study, dietary exposure was assessed using a validated 147-item semi-quantitative food frequency questionnaire (FFQ). Dietary patterns were derived through LCA. Binary logistic was performed to test the associations of dietary patterns with VAI, TyG, inflammation biomarkers, and body composition. Health centers in Tehran, Iran. 376 obese and overweight women, aged > 18 years. Two dietary patterns were identified using LCA modeling: healthy and unhealthy. Women in the unhealthy class were characterized by higher consumption of fast food, sweetened beverages, grains, unhealthy oils, butter and margarine, and snacks. Compared with the healthy class, the unhealthy class was associated with an increased risk of higher fasting blood sugar (FBS) (OR = 6.07; 95% CI: 1.33–27.74, P value = 0.02), c-reactive protein (CRP) (OR = 1.72; 95% CI: 1.05–2.80; P value = 0.02), and lower fat free mass index (FFMI) (OR = 0.56; 95% CI: 0.35–0.88, P value = 0.01), after adjusting for confounders. We found that adherence to an unhealthy dietary pattern was associated with decreased FFMI and increased FBS and CRP using LCA, but not with the rest of the variables. Further studies should be conducted to confirm the veracity of these findings.

## Introduction

Epidemiological studies highlight that the prevalence of overweight and obesity, especially abdominal obesity, is increasing worldwide^1^. In developing countries, like Iran, abdominal obesity prevalence was reported to be 76.4%, and it seems that this type of obesity is more common in women (81.4%) than in men (68.6%)^2^. Despite its prevalence, abdominal obesity is not well understood, treated, or managed among Iranians^3^. As a result, early and precise assessment of obesity-related risk factors is crucial for effectively lowering the disease burden and ameliorating its incidence^4^. Additionally, there has been a growing interest in combining biochemical and anthropometric parameters, leading to the creation of indices like the visceral adiposity index (VAI), fat-free mass index (FFMI), and triglyceride-glucose index (TyG), as alternatives to expensive methods for imaging and invasive diagnostic procedures that expose individuals to high amounts of radiation, making them more cost-effective choices^5^.

Abdominal obesity, also referred to as visceral obesity, has a strong link to mild systemic inflammation and a remarkable risk of metabolic syndrome (MetS), cardiovascular disease (CVA), type 2 diabetes (T2DM), and certain types of cancer^6–8^. The visceral adiposity index (VAI), a novel index, assesses abdominal fat distribution and dyslipidemia by combining anthropometric and laboratory parameters such as waist circumference, body mass index, circulating triglycerides, and high-density lipoprotein (HDL) cholesterol^6,9^. Based on previous research, VAI seems to represent a better predictive tool than common clinical parameters for metabolic disorders in Chinese and Caucasian samples^5,10^. Considering the various characteristics of body fat in different populations^11^, it is important to conduct more studies in this context. In addition, the Triglyceride-Glucose Index (TyG) is another new marker that is derived from triglyceride levels and fasting blood glucose levels^5^. Based on previous observational studies, TyG is not only a significant indicator of insulin resistance and diabetes risk, but also is associated with metabolic and cardiovascular disorders^10,11^. Fat-free mass index (FFMI) is another body composition index that compute fat-free mass divided by the height squared and it can modify the differences of body fat percentages related with height^12^. So, VAI, TyG and FFMI have been identified as great predictor markers for CVA, T2MD, and MetS screening in high-risk populations, such as overweight or obese women with a higher prevalence of abdominal obesity in Iran^9,11–13^. Existing evidence suggests that lifestyle modification and dietary intake affect visceral fat tissue (VAT)^14^. For example, the results of a previous study indicate that higher dietary protein intake and animal-derived monounsaturated fatty acids may be positively linked to visceral fat dysfunction and changes in VAI^12^. Contrary to the aforementioned results, another study did not detect any significant association between western, healthy, and mixed pattern with TyG and visceral level fat, even after controlling for confounders^11^. A recent Iranian-based study found that total protein replacement with carbohydrates was positively correlated with VAI, exclusively in women^15^. Moreover, results of a study on 498 overweight participants with abdominal obesity found that the Western diet pattern was negatively correlated with FFMI, and that people who were adherent to a Mediterranean diet pattern had lower FMIs, visceral fat areas, and FMI/FFMI ratios^12^. However, there was no difference in FFMI across children from both dietary patterns (health-conscious vs. sweet and processed) in the other research^16^.

On the other hand, excessive VAT induces inflammation by increasing the release of proinflammatory cytokines, which can, in turn, be measured by using serum inflammatory biomarkers like C-reactive protein (CRP)^17^. Therefore, the assessment of body composition along with dietary patterns can be useful for predicting modifications in health and disease^13^. However, it is important to point out that food ingredients and nutrients are consumed in combination, and a combination of nutrients has a different impact on health outcomes compared to a single nutrient^12,18^. In this regard, analysis of dietary patterns' effects, instead of a single-nutrient approach, on disease risk has gained prominence^12^. Various methods are available, and still being developed, for determining dietary patterns in different populations^19^. Latent class analysis (LCA), as a person-centered and model-based cluster analysis approach, recognizes the pattern of relationships across a collection of observed variables and categorizes homogeneous individuals into particular latent classes^20^. Therefore, in each class, the subjects are highly similar while they are different from the members of other classes^11^. One advantage of the aforementioned analysis is the ability to control for other variables, such as confounders, when identifying dietary patterns^15^. BThe molecular mechanisms by which dietary patterns influence the risk of chronic conditions are not completely known, however, there is reasonable evidence suggesting that a combination of nutrients, instead of individual nutrients, will affect the risk. Consequently, a pattern of nutrients may reveal more details about the most likely underlying mechanisms^21^. Notably, cultural, demographic, and economic variations, as well as differences in the study populations and regions, may have a significant impact on the outcomes^11^. As a result, it can be challenging to reconcile conflicting data in the literature, which emphasizes the necessity of further study. According to this, the purpose of the present study is to investigate, for the first time, the association between dietary patterns derived through latent class analysis (LCA) with visceral adiposity index (VAI), Triglyceride-Glucose Index (TyG), inflammation biomarkers, and body composition in overweight and obese Iranian women.

## Subjects and methods

### Study population

Three hundred and seventy-six overweight or obese women took part in this cross-sectional study. The ethics committee of Tehran University of Medical Sciences (TUMS) in Tehran, Iran, reviewed and approved the written informed consent of all study participants. Their ages ranged from 18 to 68 years and their BMI ranged from 25 to 40 kg/m^2^. The exclusion criteria were diabetes types I and II, cardiovascular disease (CVD), malignancies, liver and kidney disease, thyroid disease, menopause, pregnancy, lactation, smoking, any acute and chronic diseases, taking supplementation for weight loss, dieting during the last year, and receiving lipid, glucose, and blood pressure lowering drugs.

### Anthropometric measurements and body composition

A calibrated digital scale was used to evaluate the body weight of each subject, to the nearest 100 g, while they were barefoot and wearing light clothing. The height of the participants was measured using a non-elastic tape. To the nearest 0.5 cm, while they were asked to stand next to the wall and unshod. For calculating BMI (in square meters), the weight (in kilograms) was divided by the square of the height. To measure waist circumference elastic measuring tape was placed at the narrowest part of the waist, with a precision of 0.5 cm. Hip circumference evaluation was performed using strapless tape at the most prominent circumference. Each measurement was performed by one person to reduce possible measurement errors. A bioelectrical impedance analyzer (BIA) (Inbody 770 Co., Seoul, Korea) was used to measure all participant's body composition, and operated based on manufacturer's guidelines^12^. The subjects stood on the balance scale foot pad while holding the BIA handles with no shoes and any metal items or extra clothes. Sex, age, and height were entered into the device program. BIA evaluation took about 1 min and then data was reported as weight, skeletal muscle mass, fat-free mass, fat mass, visceral fat, body fat percentage, bone mineral content, and limb skeletal muscle mass.

### Assessment of physical activity

We measured physical activity based on the short-form of the International Physical Activity Questionnaire (IPAQ). A validity and reliability assessment of IPAQ questionnaires was conducted across 12 countries to calculate all participants' physical activity over the past 7 days. These questionnaires had high criterion reliability, with a Spearman's ρ correlation score of 0.8 and a median validation score of 0.30, which was similar to other validation studies. IPAQ as a validated self-report tool that estimates physical activity levels during the last week, was used in the current study^16^.

### Biochemical and hormonal determination

We collected venous blood between 8:00 a.m. and 10:00 a.m. after overnight fasting. We centrifuged the serum, aliquoted it, and stored it at –80 °C. We analyzed the sample with a single assay technique. We measured fasting blood glucose (FBS), triglyceride (TG) and total cholesterol (TC) by using glucose oxidase-phenol 4-aminoantipyrine peroxidase (GOD-PAP) and glycerol-3-phosphate oxidase–phenol 4-aminoantipyrine peroxidase (GPOPAP) enzymatic endpoints, respectively. Low-density-lipoprotein (LDL) and high-density lipoprotein (HDL) cholesterol were measured by using a direct enzymatic clearance assay. We assessed serum inflammatory markers via an immunoturbidimetric assay (high sensitivity assay, Hitachi 902). We used the Randox Laboratories (Hitachi 902) kit for all measurements and assessed all samples by standard methods at the Nutrition and Biochemistry Laboratory of the School of Nutritional and Dietetics at TUMS.

### Dietary intake assessment

A validated 147-item semi-quantitative food frequency questionnaire (FFQ), that had previously been validated for reliability and validity, was used to assess dietary exposure^17^. In the presence of a dietitian, participants recorded their consumption frequency in grams and milliliters based on their usual diet. Utilizing the NUTRITIONIST 4 (First Data Bank, San Bruno, CA) food analyzer, dietary intake was analyzed for energy intake, macronutrients, and micronutrients^22^.

### Dietary patterns derived by LCA

**Figure 1:**
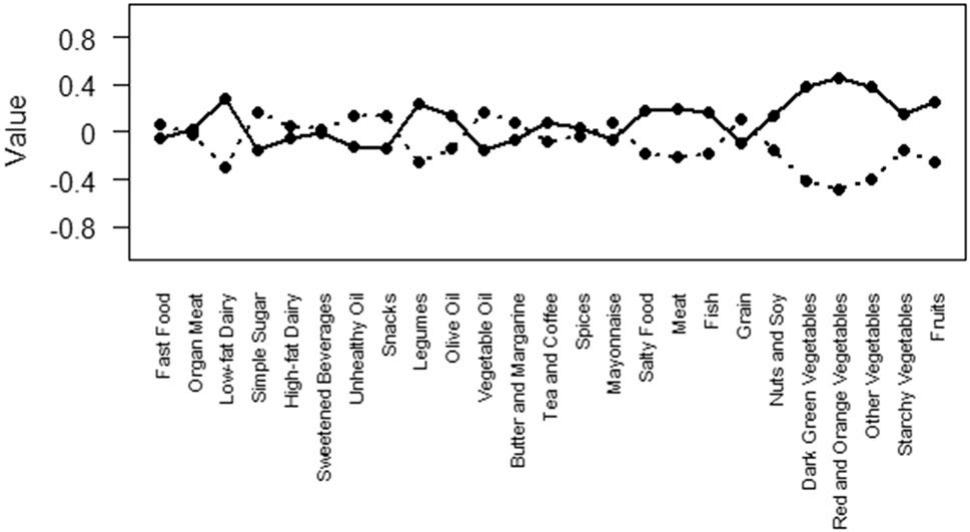
Item probabilities in the two latent classes (straight line: healthy dietary pattern, dotted line: unhealthy dietary pattern).

LCA would seek groups of subjects (classes) whom follow a common dietary pattern that is distinguishable from other groups. To determine dietary patterns, all reported food items were divided into 25 different food groups. In order to perform the latent class analysis, we first categorized people according to their energy intake according to Dietary Guidelines for Americans 2020–2025 (DGA). Following this, as LCA applies to categorized variables, individuals in each category of energy intake were split into two groups: those who consumed less than daily recommendations and those who consumed more than daily recommendations. The cut-off points for consumption categories for some food groups (legumes, meat, fish, grain, dark green, red, and orange vegetables, starchy vegetables, other vegetables, fruits, nuts, and soy) were established in accordance with the Dietary Guidelines for Americans (DGA)^23^. For other food categories for which there was no cut-point based on DGA, individuals were split into two groups based on median intake. As the amount of daily intake in different food groups can be completely different (e.g., considering spice consumption in grams compared to those of fruits), we first standardized all the variables. Then, we applied different LCA models to the data based on the different classes. Models were compared to each other using four criteria, including AIC, BIC, G^2^, and χ^2^. Based on the results, an LCA with two classes was selected as the final model. So, having the estimated classes identified, we tracked the exact pattern of consumption of a general person in each class. The average daily intake of 25 food groups was computed separately for each class. Eventually, the presence of a lower average daily intake in some food groups and a higher average in others resulted in the inference that the LCA1 class was categorized as "unhealthy", whilst the LCA2 class, with a higher average daily intake compared to one of the other classes, was classified as a "healthy" dietary pattern (Fig. 1).

### Definitions

The VAI and TyG index were calculated using the following^18,24^:

$$\mathrm{TyG\,\, index}= Ln\left[\frac{\mathrm{fasting\,\, TG }(\mathrm{mg}/\mathrm{dL}) \times \mathrm{ glucose }(\mathrm{mg}/\mathrm{dL})}{2}\right]$$

$$\mathrm{VAI\,\,women }=\left[\frac{WC}{36.58+1.89*BMI}\right]*\left[\frac{TG}{0.81}\right]*\left[\frac{1.52}{HDL-C}\right]$$

### Statistical analysis

Dietary patterns were obtained using the poLCA (version 1.6.0.1) package from R software (R-4.2.1). To determine the normality of data distribution, the Kolmogorov–Smirnov test was used; quantitative data were reported as means and standard deviation (SD), and categorical data were reported as numbers with percentages. General linear models [i.e., Analysis of variance (ANOVA) and analysis of covariance (ANCOVA)] were built to compare the body composition, VAI, TyG, and inflammatory profile between subjects. Binary logistic regression was used to determine whether an unhealthy dietary pattern was associated with cardiometabolic risk factors.

To use binary logistic regression some quantitative outcomes converted to categorical according to Karelis criteria, that was defined as follows: TG ≤ 1.7 mmol/L, HDL ≥ 1.3 mmol/L, LDL ≤ 2.6 mmol/L and, hs-CRP ≤ 3.0 mg/L, and HOMA-IR ≤ 2.7^25^. BMI was classified as overweight (BMI = 25–29.99) and obese (BMI = 30–40). WC, WHR, and FBS were categorized as ≥ 95 cm^26^, ≥ 0.85^27^, and 110 mg/dl^28^ respectively. For variables which a specific cut-off point was not defined, their median was used for classification.

In adjusted model 1, age, BMI, and energy intake were controlled. In adjusted model 2, age, BMI, energy intake, education, job, and physical activity were controlled. An odds ratio (OR) with a 95% Confidence Interval (CI) was calculated. Statistical analysis was performed using SPSS v.26 software (SPSS Inc., IL, USA). Statistical significance was accepted at P < 0.05, while P = 0.05 was considered marginally significant in the present study.

### Ethical standards disclosure

The present study was carried out in accordance to the ethical standards laid down in the 1964 Declaration of Helsinki. This investigation was also approved by the Ethics Committee of Tehran University of Medical Sciences, Tehran, Iran (with ethics number: IR.TUMS.MEDICINE.REC.1400.1515). All of the study participants signed a written consent form related to this study. Each individual was informed completely regarding the study protocol and provided a written and informed consent form before taking part in the study. literate family members of illiterate participants provided informed consent for the study and this method is approved by the Ethics Committee of Tehran University of Medical Sciences, Tehran, Iran.'

## Results

### Study population characteristics

Three hundred and seventy-six participants completed this study. The mean (SD) age and BMI of participants were 36.67 (9.21) years and 31.01 (3.85) kg/m^2^, respectively. 107 (28.4%) respondents had a moderate economic status, and the majority of respondents had a diploma [144 (38.2%)] or bachelor's degree or higher [182 (48.3%)].

### Dietary patterns derived by LCA

Latent class models were fitted for 2 to 6 classes, and finally, two dietary pattern classes were chosen. Bayesian Information Criteria, among other model diagnostics, indicated that two classes were optimum(two classes, BIC = 10,964.66; three classes, BIC = 11,019.34; four classes, BIC = 11,094.86; five classes, BIC = 11,191.17; six classes, BIC = 11,287.96). The proportion of each class, after dividing into two categories, was also balanced. Therefore, based on the balance of statistical fit and parsimony, we concluded that the 2-class model is appropriate. Figure 1 shows the conditional probabilities of participants taking each food group in each class. We named the two chosen classes as "healthy dietary pattern" and "unhealthy dietary pattern” Each line show the food consumption of participant from each dietary pattern. Continuous line indicates healthy dietary pattern and dotted line shows unhealthy dietary pattern.

Table 1 presents the mean consumption of food groups in each class. The dietary pattern had a higher percentage of individuals with a higher consumption of all kinds of vegetables, fruit, low-fat dairy, meat, fish, olive oil, vegetable oil, legumes, tea and coffee, nuts, and soy. This pattern was thus labelled a “healthy dietary pattern”. On the other hand, another dietary pattern presented the higher frequency of people with a higher consumption of fast food, sweetened beverages, grains, unhealthy oil, butter and margarine and snacks. This pattern was labelled as an “Unhealthy dietary pattern”. Overall, by conditional probability, 48.5% and 51.2% of women were characterized by unhealthy dietary pattern and healthy dietary pattern respectively.

**Table 1:** Average intake of food groups for healthy and unhealthy dietary pattern (UDP) in obese and overweight women (n = 376).

Food group	Food group components	UDP	HDP
Fast food	Pizza and sausages	22.42	19.37
Organ meat	Internal organs of lamb such as the liver, heart, kidney	2.27	2.48
Low-fat dairy	Low fat milk, cheeses, low fat yogurt	181.15	300.02
High-fat dairy	High fat milk, cream, high fat yogurt, high fat cheeses, strained yogurt	95.92	81.64
Simple sugar	Biscuits, crackers, cakes, sugar, candy, chocolate, honey, soft drinks, jam, and all kind of sweets	48.64	35.97
Snacks	Chips and corn puffs	10.87	6.79
Sweetened beverages	Commercially produced fruit juices	45.63	41.73
Unhealthy oil	Hydrogenated oil, animal oil	11.18	6.80
Vegetable oil	Vegetable oil	15.61	11.25
Olive oil	Olive oil, olives	1.81	3.11
Legumes	Beans, peas, lentils, mung beans, chickpeas, beans	37.80	57.48
Butter and margarine	Butter and margarine	4.90	3.79
Tea and coffee	Tea and coffee	681.37	807.67
Spices	All kind of spices	3.23	3.41
Salty food	Salt, pickles	58.56	82.97
Mayonnaise	Mayonnaise	2.46	2.00
Meat	Beef, lamb, eggs, and poultry	71.81	91.88
Fish	Fish and canned tuna fish	5.82	9.22
Grain	All kind of bread, rice, pasta, noodles, vermicelli, wheat flour, barley, oatmeal, and corn	469.00	426.76
Dark green vegetables	Spinach, leafy greens, and lettuce	40.93	83.55
Red and orange vegetables	Carrots, tomatoes, pumpkin	94.17	204.14
Starchy vegetables	Green peas, potatoes	23.13	31.29
Other vegetables	Cucumbers, celery, green beans, green peppers, bell peppers, turnips, zucchini, pumpkin, mushrooms, onion, garlic, any kind of cabbage	138.67	257.28
Fruits	Apples, cherries, apricots, plums, figs (dried or fresh), kiwi, strawberries, grapes or raisins, dates, bananas, pomegranates, melons, oranges, tangerines, grapefruits, pears, persimmons, cantaloupe, melons, watermelons, nectarines, peaches, greengage, lemons, berries (dried or fresh), and other dried fruits, orange juice, apple juice, cantaloupe juice and fruit compote	414.66	565.92
Nuts & soy	Almonds, peanuts, walnuts, pistachios, hazelnuts, seeds, and soy	16.73	23.42

Values are represented as means (SD).

Average consumption is reported in grams.

*UDP* unhealthy dietary pattern, *HDP* healthy dietary pattern, *SD* standard deviation.

The energy intake of women characterized by an unhealthy dietary pattern and a healthy dietary pattern was 2576.25 kcal/day and 2676.67 kcal/day, respectively.

Sociodemographic characteristics, visceral adiposity index (VAI), triglyceride-glucose index (TyG), biochemical variables, and body composition according to Dietary Patterns.

The mean and SD of sociodemographic characteristics, visceral adiposity index (VAI), triglyceride-glucose index (TyG), biochemical variables, and body composition of subjects according to their dietary patterns are shown in Table 2. The “unhealthy dietary pattern” presented a lower age mean compared to the “healthy dietary pattern” (p = 0.008). In addition, physical activity was significantly lower among those with an unhealthy dietary pattern (p = 0.003). After adjusting for confounders such as age, BMI, physical activity, education, job, and energy intake, there was no significant difference between the two dietary patterns in other variables.

**Table 2:** Mean and SD of sociodemographic characteristics, visceral adiposity index (VAI), triglyceride-glucose index (TyG), biochemical variables, and body composition in obese and overweight women (n = 376).

Variables^†^	Dietary patterns					
	UDP, N = 183		HDP, N = 193		P-value	P-value*
	Mean or N	SD or %	Mean or N	SD or %		
Demographic characteristics						
Age (years)	35.44	9.40	37.92	8.84	**0.009**	**0.008**
IPAQ (MET min-week)	830.76	803.65	1152.77	13.09	**0.007**	**0.003**
Economic category						
Poor	10	50	10	50	0.98	
Moderate	52	49.1	54	50.9		
Good	153	48.7	161	51.3		
Education category						
Illiterate	2	50.0	2	50.0	0.99	
Under diploma	21	46.7	24	53.3		
Diploma	71	49.3	73	50.7		
Bachelor and higher	87	48.1	94	51.9		
Job						
Housekeeper	101	46.8	115	53.2	0.26	
Worker	4	66.7	2	33.3		
Home job	8	50	8	50		
Employee	29	55.8	23	44.2		
Self-employment	29	43.9	37	56.1		
Student	13	68.4	6	31.6		
BMI category						
Overweight	85	51.5	80	48.5	0.35	
Obese	99	46.7	113	53.3		
VAI	2.19	1.53	2.68	2.50	0.08	0.13^a^
TyG	8.38	0.51	8.48	0.52	0.13	0.18
Anthropometric and body composition measurements						
Body weight (kg)	80.18	11.25	81.10	11.43	0.43	0.61
Height	161.40	5.98	161.02	5.59	0.51	0.75
BMI (Kg/m^2^)	30.82	3.85	31.21	3.87	0.31	0.60
WC (cm)	99.03	9.79	99.36	9.44	0.73	0.95^a^
WHR (ratio)	0.93	0.05	0.93	0.05	0.65	0.95^a^
BFM (kg)	34.20	8.04	34.37	8.02	0.83	0.87^a^
FFMI	17.65	1.51	18.65	9.50	0.16	0.38^a^
FMI	13.18	3.10	13.35	3.13	0.60	0.86^a^
Biochemical variables						
FBS (mg/Dl)	87.97	10.82	86.75	8.77	0.34	0.15
Total cholesterol (g/dl)	182.49	37.32	184.26	34.67	0.70	0.53
TG (mg/dl)	111.37	56.33	128.33	77.89	0.06	0.24
HDL (mg/dl)	47.58	10.04	45.83	10.95	0.21	0.21
LDL (mg/dl)	94.59	24.47	93.77	23.39	0.79	0.60
hs-CRP (mg/L)	5.46	4.74	4.69	4.38	0.10	0.07

Values are represented as means (SD).

Categorical variables: N and %.

ANCOVA (P value*) was performed to adjust potential confounding factors (age, BMI, energy intake, physical activity, education, job).

p-values < 0.05 were considered significant.

*UDP* unhealthy dietary pattern, *HDP* healthy dietary pattern, *IPAQ* International Physical Activity Questionnaire, *VAI* visceral adiposity index, *TyG* triglyceride-glucose index, *BMI* body mass index, *WC* waist circumference, *WHR* waist to hip ratio, *BFM* body fat mass, *FFMI* fat-free mass index, *FFM* fat-free mass, *FMI* fat mass index, *HDL_C* high-density lipoprotein cholesterol, *LDL_C* low-density lipoprotein cholesterol, *TG* triglyceride, *hs CRP* high-sensitivity C-reactive protein.

^a^BMI is considered a collinear variable for anthropometrics and body composition variables.

Significant values are in bold.

### Association between dietary patterns and visceral adiposity index (VAI), triglyceride-glucose index (TyG), biochemical variables, and body composition

Crude and adjusted OR and 95% CI of variables across dietary patterns were shown in Table 3.

**Table 3:** Binary logistic regression analysis of the association between dietary patterns and visceral adiposity index (VAI), triglyceride-glucose index (TyG), biochemical variables, and body composition in obese and overweight women (n = 376).

Variables	HDP, N = 193	UDP, N = 183		P-value
		Odds ratio	95% confidence interval	
Anthropometric and body composition measurements				
BMI				
Crude	1.00 (reference)	0.83	(0.55, 1.25)	0.38
Model 1	1.00 (reference)	0.93	(0.61, 1.41)	0.74
Model 2	1.00 (reference)	0.86	(0.54, 1.37)	0.53
WHR				
Crude	1.00 (reference)	0.99	(0.66, 1.50)	0.98
Model 1	1.00 (reference)	1.02	(0.67, 1.55)	0.91
Model 2	1.00 (reference)	0.97	(0.62, 1.52)	0.90
BFM				
Crude	1.00 (reference)	0.88	(0.59, 1.33)	0.56
Model 1	1.00 (reference)	0.96	(0.63, 1.45)	0.84
Model 2	1.00 (reference)	0.91	(0.58, 1.44)	0.71
WC				
Crude	1.00 (reference)	0.81	(0.54, 1.22)	0.32
Model 1	1.00 (reference)	0.86	(0.57, 1.30)	0.47
Model 2	1.00 (reference)	0.81	(0.52, 1.27)	0.37
FFMI				
Crude	1.00 (reference)	0.58	(0.38, 0.87)	**0.009**
Model 1	1.00 (reference)	0.63	(0.41, 0.96)	**0.03**
Model 2	1.00 (reference)	0.56	(0.35, 0.88)	**0.01**
FMI				
Crude	1.00 (reference)	0.80	(0.53, 1.21)	0.29
Model 1	1.00 (reference)	0.89	(0.59, 1.36)	0.61
Model 2	1.00 (reference)	0.86	(0.54, 1.35)	0.51
VAI				
Crude	1.00 (reference)	0.60	(0.35, 1.01)	**0.057**
Model 1	1.00 (reference)	0.63	(0.37, 1.07)	0.09
Model 2	1.00 (reference)	0.62	(0.34, 1.13)	0.12
TyG				
Crude	1.00 (reference)	0.66	(0.37, 1.17)	0.16
Model 1	1.00 (reference)	0.78	(0.43, 1.40)	0.40
Model 2	1.00 (reference)	0.81	(0.43, 1.52)	0.52
Biochemical variables				
FBS				
Crude	1.00 (reference)	3.16	(1.06, 9.42)	**0.03**
Model 1	1.00 (reference)	4.72	(1.48, 15.06)	**0.009**
Model 2	1.00 (reference)	6.07	(1.33, 27.74)	**0.02**
TG				
Crude	1.00 (reference)	1.40	(0.76, 2.59)	0.27
Model 1	1.00 (reference)	1.58	(0.84, 2.98)	0.15
Model 2	1.00 (reference)	1.71	(0.85, 3.42)	0.12
LDL				
Crude	1.00 (reference)	1.42	(0.83, 2.44)	0.19
Model 1	1.00 (reference)	1.78	(1.00, 3.18)	**0.04**
Model 2	1.00 (reference)	1.54	(0.82, 2.88)	0.17
HDL				
Crude	1.00 (reference)	0.72	(0.41, 1.25)	0.24
Model 1	1.00 (reference)	0.66	(0.37, 1.17)	0.16
Model 2	1.00 (reference)	0.67	(0.36, 1.25)	0.21
hsCRP				
Crude	1.00 (reference)	1.39	(0.92, 2.09)	0.11
Model 1	1.00 (reference)	1.45	(0.94, 2.25)	0.09
Model 2	1.00 (reference)	1.72	(1.05, 2.80)	**0.02**

Binary logistic regression was used.

A healthy dietary pattern considers a reference group.

Data are presented as odds ratio (OR) and (95% confidence interval).

P-values < 0.05 were considered significant.

P-values = 0.05 were considered marginally significant.

P value with unadjusted (crude).

Adjusted model 1: adjusted for age, energy intake, BMI.

Adjusted model 2: adjusted for age, energy intake, BMI, physical activity, education, job.

*UDP* unhealthy dietary pattern, *HDP* healthy dietary pattern, *VAI* visceral adiposity index, *TyG* triglyceride-glucose index, *BMI* body mass index, *WC* waist circumference, *WHR* waist to hip ratio, *BFM* body fat mass, *FFMI* fat-free mass index, *FFM* fat-free mass, *FMI* fat mass index, *HDL_C* high-density lipoprotein cholesterol, *LDL_C* low-density lipoprotein cholesterol, *TG* triglyceride, *hs CRP* high-sensitivity C-reactive protein.

Significant values are in bold.

A healthy dietary pattern was used as a reference. Binary logistic analysis showed that the unhealthy dietary pattern class was associated with an increased risk of higher FBS and lower FFMI in the crude model (P < 0.05). After adjustment with confounders in model 1 (adjusting for age, energy intake, and BMI), participants with unhealthy dietary pattern had lower odds of FFMI (OR = 0.99; 95% CI: 0.35–2.81; P value = 0.040), and higher odds of FBS and LDL (OR = 0.99; 95% CI: 0.35–2.81; P value = 0.040). In the fully adjusted model (adjusting for age, energy intake, BMI, education, job, and physical activity), we found that an unhealthy dietary pattern was strongly associated with an increased risk of higher FBS (OR = 6.07; 95% CI: 1.33–27.74, P value = 0.02), lower FFMI (OR = 0.56; 95% CI: 0.35–0.88, P value = 0.01), and significant associated with an increased risk of higher CRP (OR = 1.72 95% CI: 1.05–2.80; P value = 0.02). No significant relationship between unhealthy dietary pattern and other outcomes was observed.

## Discussion

In the current study, for the first time, we applied LCA modelling to investigate the association between dietary patterns (DPs) with VAI, TyG index, inflammation, and body composition in an overweight and obese Iranian female population. We found that following an unhealthy dietary pattern, characterized by high intakes of fast food, sweetened beverages, grain, unhealthy oil, butter, margarine, and snacks, was associated with lower FFMI and a higher risk of increased FBS and CRP compared to a healthy dietary pattern, with a high load of vegetables, fruit, low-fat dairy, meat, fish, olive oil, vegetable oil, legumes, tea and coffee, nuts, and soy. Moreover, we did not detect any significant association with the rest of the variables in the fully adjusted model.

Diet acknowledgedly p lays an important role in the development of insulin resistance, in particular glucose serum levels^29,30^, and the potential contributions of diet to body composition, inflammation, VAI, and TyG index have also been reported^11,21,31,32^. A study among medical university students in China that examined the impact of different dietary pattern on body composition showed that the Western pattern influenced FMI/FFMI ratio positively, while the "vegetable and fruit" pattern influenced FMI/FFMI ratio negatively^33^. Similarly, another study in 6-year-old children proposed that high intake of fruit, vegetables, grains, and vegetable oils can result in elevated FFMI^34^. Findings from a meta-analysis that assessed the effects of saturated fatty acid (SFA), polyunsaturated fatty acid (PUFA), monounsaturated fatty acids (MUFA), and carbohydrates on glucose-insulin homeostasis revealed that only the substitution of energy intake with PUFA was related to lower FBS^35^. This significant association may be related to the potential health benefit of PUFA in suppressing oxidative stress and insulin resistance^36^. Another study in participants with the highest category of whole-grain consumption demonstrated lower levels of CRP and FBS, compared with participants in the lowest category of whole-grain consumption^8^. One publication, among adult Americans, indicated positive associations between VAI and glucose/insulin homeostasis markers with a higher dietary proportion of carbohydrate and sugar, total fat, and SFA, and negative associations with a diet consisting of fiber, vitamins, and minerals. An inverse association was also found between a diet rich in PUFA and MUFA with FBS^37,38^. In support of our findings, a five-year prospective study failed to find an association contributed to PUFAs, MUFAs, and SFAs with changes in VAT^39^. There was also no significant relationship between healthy dietary pattern with visceral fat level and TyG index^40^. However, in a study conducted on Iranian adults, where individuals followed a diet rich in MUFA concomitant to decreased dietary intake of total protein or PUFA, a positive association with VAI changes was observed^41^. More so, a cross-sectional study revealed a significant direct association between fat intake and visceral adipose tissue (VAT) in overweight young adults^40^. Another study among older Americans reported a negative association between the Dietary Approaches to Stop Hypertension (DASH) diet index and VAI^42^. In addition, in a prospective cohort study, an inverse association between anti-inflammatory diet and TyG index was shown^43^.

In comparison to following a healthy dietary pattern, 6–13-year-old children who followed a Western pattern had higher levels of glucose and LDL and lower levels of HDL^44^. An investigation among Brazilian adults exhibited an inverse association between following a healthy dietary pattern with obesity-related markers (BMI, WC, and WHR), FBS, TG/HDL, and LDL/HDL values, and a positive association with HDL. In contrast, adherence to the "Traditional" pattern was positively associated with BMI, WC, and WHR and had a significant inverse association with body fat, LDL, HDL, and TyG^45^. Notwithstanding an inverse association between adherence to a traditional dietary pattern and excess body fat, no relationship was detected between the healthy, snack, and unhealthy dietary patterns with obesity or body adiposity^46^.

The putative mechanism accounting for the obtained results of following a healthy dietary pattern is that the higher fiber content of vegetables, legumes, and nuts, can lead to lower nutrient absorption or energy reduction, and therefore affects total fat mass and visceral fat accumulation. Furthermore, higher fiber consumption displayed tendencies toward improved insulin resistance and reduced visceral fat adiposity. It has been demonstrated that low glycemic index carbohydrates from vegetables, legumes, and nuts can also decrease insulin resistance^41,47,48^. On the other hand, lower systemic inflammation may be related to the anti-inflammatory and anti-oxidative properties of olive oil, nuts, soy foods, fiber, and legumes^40,49^. Consequently, the mechanism behind the effects of unhealthy dietary pattern on the aforementioned markers might be attributed to an imbalance in diet adherence with low levels of certain vital nutrients that are prone to inflammation, increased FBS, and decreased FFMI^43^. For instance, higher sugar beverage consumption with high amounts of rapidly absorbable carbohydrates can lead to enhanced FBS and insulin resistance^50^. Comparisons of our findings with previous reports are difficult to make due to the different approaches applied to specifying dietary patterns. Nevertheless, these results highlight the importance of following a healthy dietary pattern to ameliorate the above-mentioned markers.

LCA is a robust, data-driven, analytic approach that considers the source of heterogeneity from participants of a studied population instead of diet measurement variables. This person-centered novel model may be more effective at identifying dietary patterns when compared to previous models^51,52^.

To the author's knowledge, there are no comparable studies that have been conducted in developing countries that have jointly investigated the association between dietary patterns and VAI, TyG index, inflammation, and body composition using LCA. Given that participants use a combination of foods rather than a single food, evaluating dietary patterns to examine the mentioned associations provides accurate information and should be considered as an additional strength. However, there are several limitations to this study that need to be noted. Due to the cross-sectional nature of the study, we could not draw any causal inferences. In addition, the study relied on self-reported dietary data, and reporting inaccuracies are expected in this regard. The present study, with a small sample size, included only obese women, and therefore the results cannot be extrapolated to all populations.

## Conclusion

In conclusion, we found that following an unhealthy dietary pattern was associated with higher blood levels of FBS and CRP and lower FFMI using LCA, but not with the rest of the variables. To confirm these findings, focusing on large prospective studies in different populations is required.

## Data Availability

The datasets generated and/or analysed during the current study are available in the Khadijeh Mirzaei repository. The corresponding author should be contacted if someone wants to request the data from this study.
